# Discrete Model of Opinion Changes Using Knowledge and Emotions as Control Variables

**DOI:** 10.1371/journal.pone.0044489

**Published:** 2012-09-11

**Authors:** Pawel Sobkowicz

**Affiliations:** KEN 94/140, Warsaw, Poland; Cinvestav-Merida, Mexico

## Abstract

We present a new model of opinion changes dependent on the agents emotional state and their information about the issue in question. Our goal is to construct a simple, yet nontrivial and flexible representation of individual attitude dynamics for agent based simulations, that could be used in a variety of social environments. The model is a discrete version of the cusp catastrophe model of opinion dynamics in which information is treated as the normal factor while emotional arousal (agitation level determining agent receptiveness and rationality) is treated as the splitting factor. Both variables determine the resulting agent opinion, which itself can be in favor of the studied position, against it, or neutral. Thanks to the flexibility of implementing communication between the agents, the model is potentially applicable in a wide range of situations. As an example of the model application, we study the dynamics of a set of agents communicating among themselves via messages. In the example, we chose the simplest, fully connected communication topology, to focus on the effects of the individual opinion dynamics, and to look for stable final distributions of agents with different emotions, information and opinions. Even for such simplified system, the model shows complex behavior, including phase transitions due to symmetry breaking by external propaganda.

## Introduction

The goal of opinion research is to identify trends in public attitudes, shifts of views, and expectations of stakeholder groups and reactions to specific events or policies. A major application of opinion research is the area of anticipation and prediction of impacts of policy measures and improvement of communications towards the desired goals. Models of opinion formation based on real-world online communication enable the simulation and prediction of the evolution of attitudes. Opinion research is not limited to traditional tools, such as dedicated polls. The ubiquitous computer based communications provide large amounts of data, from which it is possible to observe and study social interactions and opinions. The Internet has been compared to a huge social and psychological laboratory [1], which might offer insights on a scale unavailable for the traditional research environments.

Recent years have brought significant interest in interdisciplinary studies, combining tools and methods known from physics with social analyses. These studies are often referred to as sociophysics, and range from purely numerical studies of economic trends to descriptions of social activities. Among the latter, a significant role is played by computational models of opinion formation [2]. Such models often use agent-based simulations. Within a simplified framework, focusing on a few selected aspects of social activities (such as communication network, susceptibility to influences, contrariness etc.), it is possible to derive general trends of behavior of large societal groups, starting from individual perspectives.

The numerical modeling of opinion changes is often based on analogy from the condensed matter physics, with opinions treated as discrete states, resembling spin states in solids. The changes of opinions in such models are typically attributed to interactions between pairs of agents, or the influence of groups of agents or external media on a single agent. The models vary considerably in the ways these interactions are described. Though simplified, these sociophysical models have shown many interesting results and are studied for more than 20 years.

There exists a variety of such models. Among the most popular, one can mention the voter model [3]–[6], the Sznajd model [7]–[14], the bounded confidence model [15]–[20], the Hegelsmann-Krause model [21], the social impact modef of Nowak-Latané [22], [23] and its further modifications including the role of leaders [24]–[27]. There are also studies comparing various models and their statistical properties for the same social networks and basic parameters, for example [2], [28]–[31] or the effects of associations of series of events and memories [32]. The common feature of these approaches is the way that opinion change is modeled, namely the dependence of the agent's changed opinion on the combination of its current opinion and the opinions of its neighbors and external influences. While the models vary in details, they often assume that the individual opinion is easily changed, provided some basic influence is present, such as the presence of one or more disagreeing agents in a neighborhood. A frequent assumption is that an agent may change its opinion after a single contact with another agent, or when perceiving that the local majority of agents favors a different opinion. These assumptions stand in disagreement with everyday observations of relative stability of individual opinions and resistance to pressures. Contacts, even frequent ones, even involving deliberative processes, only rarely lead to an opinion change. The pressure of the environment is also not a sufficient condition: being surrounded by proponents of a different view does not automatically lead to an adjustment of one's own opinion.

One of the ways of explaining the stability of opinion within the spin-like models is done by adding a sufficiently large ‘self-interaction’, which may counter the effects of external influences. The problem with such approach is that the self-influence would have to act differently, depending on social situation, adding to complexity of the models.

Another explanation of opinion stability is related to adjustments of the social network due to disagreements [27], [33]. This idea is based on an observation that a frequent reaction to profound differences in opinions between people is cutting off the social relationships between the disagreeing persons (if not excluded by specific situation, e.g. among family members or within work-teams). Cutting off awkward social links leads to simultaneous evolution of the individual opinions and the social network. A typical result is a separation of the group into a set of weakly coupled conflicted subgroups. Under such conditions, the chance to encounter people with opposing views are smaller and individual opinions are more stable even in the standard approach.

While such separation is observed in some situations, it is, however, not universal. Our motivation in providing yet another model for the opinion change process comes from our studies of Polish Internet communities, where we have observed strong relationship between user knowledge about the discussed issues, his/her emotional state and the resulting opinion dynamics [34], [35]. In contrast to the hypothesis of separation of conflicted sides, the observed communities were characterized by a dominance of contacts between the conflicted participants, with a significant amount of arguments passing between them – yet there were practically no cases of a switch of political sympathies (taken as the agent opinions). Only 15 out of over 6400 participants of the discussions changed their political party preference during a two year period. The observed stability of attitudes strongly contrasted with volatility of the emotions of the forum users.

**Figure 1:**
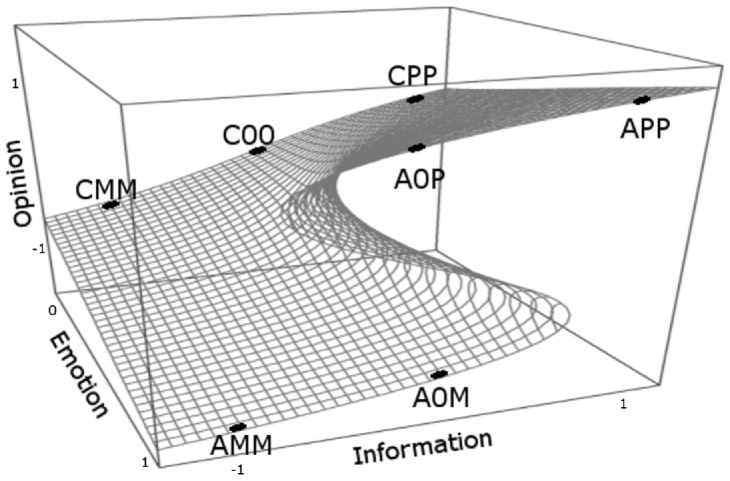
Schematic representation of the seven states of the agents, depending on emotion (*E*) and information (*I*) state, showing relationship of the current discrete model to the continuous cusp catastrophe. The two control variables are information (normal factor) and emotion (splitting factor). The states are described in the Table 3. In the agitated state (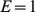) the agent may support one of the two conflicting opinions (A0M and A0P) for the same value of emotion and information. Instead of continuous paths over the cusp surface, the development of an opinion is described through jumps between the states.

This has led us to revive the approaches combining the informative and emotional influences on attitudes. One of the most developed of such approaches is the one based on the catastrophe theory. Since its introduction [36], the catastrophe theory has had its ups and downs in analyses of human behavior [37], but recent years have seen some revival. In the context of attitude change and opinion modeling, the most popular was the cusp catastrophe, which allowed to intuitively explain a hysteresis behavior [38]–[46]. The cusp catastrophe model has two independent, continuous variables generally named the normal factor and the splitting factor. The normal factor, for low values of the splitting factor, completely determines the outcome. The increase of the orthogonal splitting factor leads to appearance of a region where there are two possible outcomes for the same set of parameters (see Fig. 1). In the context of opinion change, the role of the normal factor has been variously given to information about the issue or conformity pressure. The splitting factor role was taken by the involvement in the issue, individual preconceptions or the importance of the issue for the individual. Comparing this approach with psychological literature, where literally tens of possible persuasion variables have been proposed and their importance debated [47], a two-parameter model looks enticingly simple. Still, even with a number of variables limited to two, mapping the continuous set of parameters to real life is extremely cumbersome, as shown, for example by [41], [48]–[53]. The difficulties arise from the need to precisely measure and assign numerical values to the control variables, which are often of a delicate, psychological nature: information, involvement in the issue, personal importance, emotional factors, and the difficulty of assigning numerical values with high precision.

If obtaining the values of the cusp catastrophe parameters from observations in attempt to understand specific social examples is difficult and prone to large errors, then the effective use of agent based models for predictive purposes, using the same framework, is even more difficult. We must remember that the quality of any simulation in reproducing real world data crucially depends on the ability to determine the proper input parameters, and the more errors are inherent in this choice, the more difficult is to check if `this is the right combination of parameters', corresponding to the studied social system.

In this situation we propose a simplified discrete model that includes the key property of the cusp catastrophe, that is the possibility that for certain emotional states it is possible for the agents to hold conflicting opinions despite the fact that they share the same information about the issue (corresponding to the fold in the cusp catastrophe surface). This also allows the ‘hysteresis’ behavior when the normal factor is changed continuously back and forth. The basic idea is visualized in Fig. 1, showing how the set of the discrete states describing the agent characteristics is related to the continuous surface of the traditional cusp catastrophe model.

We set out to determine if such simple model can lead to nontrivial system behavior, for example to stable co-existence of agents with different opinions and emotions – typical for the real societies and to a stability of individual opinions against external and peer pressures.

## Model Description

The model is based on a population of agents, each of them described by three parameters: opinion, information and emotion and forming a social network. We treat the opinion as dependent variable, determined by the information and emotion, which are the control variables in the parlance of the catastrophe theory. Using the information about the issue in question (which might support one of the opposing views or a balanced view) as the normal factor is a common one in the literature as it is strongly correlated with opinion within the rational deliberation regime (low values of the splitting factor), see e.g. [41], [50], [52]. On the other hand, the choice of the psychological variable for the splitting factor is less obvious. The works cited above used the involvement in the issue or the individual importance of the issue as the splitting factor. We have decided to chose emotional arousal as general description for the splitting variable, knowing that the name may have multiple meanings. Our focus is on the agent agitation or arousal level, connected to the issue in question, assuming that such arousal would influence the agent's rationality and receptiveness. We define two basic states: a calm one, in which a person accepts outside information and reasoning; and, at the same time, is capable of formulating and expressing his/her views equally reasonably. The second, agitated or excited state, is characterized by the inability to deliberate and to accept information and by a tendency to communicate in emotionally charged way, with little recourse to facts and reasoning. We understand that from the point of view of detailed psychological understanding, this is a gross simplification. For example the agitated state might influence differently the capacity to absorb information and to express one's own views. Nevertheless, we decided to unify the passive and active aspects in one measure, for the sake of model simplicity. This choice of the splitting variable, has been prompted by our observations of the emotions and opinions expressed by participants in political Internet discussions studied in [35], [54] as well as other works reporting the importance of emotions in Internet based communications [55]. Still, the choice of the best persuasion factor to play the role of the splitting factor would best be decided by psychological or even physiological experiments such as the ones reported by Walla, Brenner and Koller [56]. We note that it is likely that the involvement in the issue and the agitation level are highly correlated. We note that the opinion of an agent would depend on the messages received and on the history of its previous opinions (corresponding to the evolution path on the cusp surface in the continuous model).

In a crucial step we propose that all the variables are discretized. For example, the opinion can take three values of attitude towards certain issue: pro (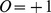), contra (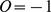), and neutral (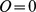). The information about the issue in question can also take three values: 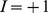, 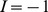 and 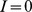. These correspond to the information supporting the above opinions. The 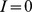 is the “uninformed” state in which the agent has no information about the issue or when the information held by the user 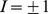 correspond to the “informed” state, where the agent has significant information supporting one of the opposite viewpoints. Lastly, the emotion arousal level is described by two values: the calm state (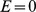) and the agitated state (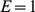). We purposefully simplify the emotion approach by focusing on the arousal level and not on the valence of the emotions. A discretized approach to emotion analysis has been suggested previously for the emotion types by Briesemeister, Kuchinke and Jacobs [57]. There are altogether seven states an agent can take located at key areas of the cusp surface (Fig. 1). These are described in the Table 3, together with the notation used in this paper. Of course the full complexity of factors influencing our opinions can not be captured in seven states. Even the full, continuous catastrophe models suffer from the limit of two-dimensional parameter space. Still, our goal was to propose a model that is as simple as possible but nontrivially extending the current single parameter approaches.

The model assumes that the agents communicate via messages, which reflect the current state of the sender of the message. Specifically, in the simplest version of the model, a message carries the same values of emotion and information as its author. This choice for the communication model is quite flexible and corresponds to many real life environments: the message may be an utterance in a conversation, an e-mail or a post in a discussion forum… The details of the model, such as the frequency of the messages, whether they are addressed to a specific person or to the general audience, probability of response, could be adjusted to cover the studied social situation. Depending on the social situation, the messages may be addressed to a particular recipient, a group or to a general audience. Also the process of receiving or ‘reading’ of the messages may be modeled to follow the actual studied situation.

The recipient of the message changes its state in response to the message, depending on the information content, emotional state of the message and the agent itself and on the expressed opinion. The rules are kept quite simple, yet aim to reflect some psychological traits of human communication. For example, with respects to emotions, an agitated message (sent by an agent with 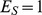) would leave agitated receiving agent (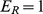) agitated. The same message, received by a calm agent would have different effects depending on the state of the recipient. For recipients who do not have an established opinion (O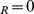), or whose opinion is the same as the one expressed by the message, the agent would stay calm. On the other hand a calm recipient with a differing opinion (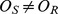) would get angered by the tone and content of the message and shift to the agitated state. Calm messages (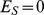) leave calm recipients calm, regardless of the possible differences in opinion. The agitated receivers, sharing the same opinion with the calm message may be assured of their opinion and therefore calm down, while if the opinions of the sender and recipient differ, the recipient would stay agitated.

The information changes of the recipient depend on the emotional state of both the sender and recipient. We assume that the information carried in the message is less than the full knowledge of the sender; we also take into account that the agitated state of any of the two agents results in weaker expression or reception of the information. As a result the information status of the recipient (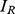) is assumed to conform to the following rules.

If both agents are calm then the influence of the information currently held by the recipient 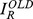 and provided by the sender of the message 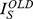 are given equal weight. The resulting new information state of the recipient 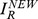 is given by Table 1.

**Table 1:** Changes of agent information state upon reception of a message in the situation when both recipient and the sender are calm.

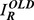 Previous recipient information	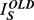 Sender information	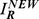 New recipient information	explanation
−1	−1	−1	no change, message confirms agent's information
−1	0	−1	no change, message contains no information
−1	1	0	change to neutral, message balances agent's information
0	−1	−1	change to negative, message “convinces” the agent
0	0	0	no change, message contains no information
0	1	1	change to positive, message “convinces” the agent
1	−1	0	change to neutral, message balances agent's information
1	0	1	no change, message contains no information
1	1	1	no change, message confirms agent's information

If at least one of the agents is in an agitated state then the influence of the information currently held by the recipient 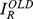 is assumed to have twice the weight than the information provided by the sender of the message 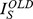. In this case, the resulting new information state of the recipient 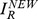 is given by the Table 2. The difference between the two cases (seen for recipients without decisive information 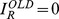), reflects our intention to capture the reluctance of uninformed, agitated people (whose opinion is, one might say, based on belief) to accept any external information. Similarly, calm recipients would give much less consideration to information contained in agitated messages.

**Table 2:** Changes of agent information state upon reception of a message in the situation when at least one of the agents (recipient and/or sender) is agitated.

Previous recipient information 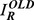	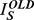 Sender information	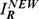 New recipient information	explanation
−1	−1	−1	no change, message confirms agent's information
−1	0	−1	no change, message contains no information
−1	1	0	change to neutral, message balances agent's information
0	−1	0	no change, message does not “convince” the agent
0	0	0	no change, message contains no information
0	1	0	no change, message does not “convince” the agent
1	−1	0	change to neutral, message balances agent's information
1	0	1	no change, message contains no information
1	1	1	no change, message confirms agent's information

**Table 3:** Possible states of agents, together with the notation used in this paper.

Symbol	Emotion	Information	Opinion
CPP	0	+1	+1
C00	0	0	0
CMM	0	−1	−1
APP	+1	+1	+1
A0P	+1	0	+1
A0M	+1	0	−1
AMM	+1	−1	−1

The first letter denotes the emotional state (calm or agitated), the second one is the information available to the agent (plus, zero or minus), the third is the agent's opinion (plus, zero or minus).

Table 4 presents the resulting states of the receiver (together with the unchanged states of the sender) for all the possible combinations.

**Table 4:** Matrix of states of agents resulting from a single message sent by the ‘Sender’ and received by the ‘Recipient’ in given state.

Recipient	Sender/Message						
	CMM	C00	CPP	AMM	A0M	A0P	APP
	CMM	C00	CPP	AMM	A0M	A0P	APP
CMM	CMM	CMM	**C00**	**CMM**	**CMM**	**AMM**	**A0M**
	CMM	C00	CPP	AMM	A0M	A0P	APP
C00	**CMM**	**C00**	**CPP**	**C00**	**C00**	**C00**	**C00**
	CMM	C00	CPP	AMM	A0M	A0P	APP
CPP	**C00**	**CPP**	**CPP**	**A0P**	**APP**	**CPP**	**CPP**
	CMM	C00	CPP	AMM	A0M	A0P	APP
AMM	**CMM**	**AMM**	**A0M**	**AMM**	**AMM**	**AMM**	**A0M**
	CMM	C00	CPP	AMM	A0M	A0P	APP
A0M	**C00**	**A0M**	**A0M**	**A0M**	**A0M**	**A0M**	**A0M**
	CMM	C00	CPP	AMM	A0M	A0P	APP
A0P	A0P	A0P	**C00**	**A0P**	**A0P**	**A0P**	**A0P**
	CMM	C00	CPP	AMM	A0M	A0P	APP
APP	**A0P**	**APP**	**CPP**	**A0P**	**APP**	**APP**	**APP**

In each cell the top is the final state of the sender (unchanged) and bottom is the state of the recipient, which may be changed. Boldface denotes changed agent states. Note that the majority of situations leave the recipient in the previous state.

The mechanism of changes of emotions and information and, subsequently, opinions described above is a fully deterministic one. It is possible, within the same basic framework to introduce non-deterministic reactions of the agents to received messages, for example by using some probability considerations related to the state changes. Two straightforward yet significant examples of such probabilistic extensions of the basic model are as follows. First, one could introduce probability 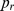, which would determine if a message would have any effect on the recipient. This would basically slow down the time evolution resulting from exchanges of messages, becoming important in studies of discussions in small groups. The second interesting introduction of randomness into the model would allow, with probability 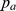, a calm agent (e.g. CPP) to react emotionally to a calm message supporting the opposite view (i.e. to a CMM message in our example), treating it as if it were an agitated one. One might call such reaction ‘allergic’. As a result instead of changing its state to C00, the recipient would change into the A0P state. The probability 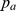 would measure the irrationality or irritability present even in the calm, deliberative and rational state. This extension would allow arousal of emotions due to disagreements even in initially calm society. This generation mechanism should then be coupled with a mechanism of gradual decrease of emotional arousal [58]–[61].

Such probabilistic extensions are fully within the framework of the proposed model, and may be crucial in realistic modeling of specific human reactions and certain environments. In such cases the relevant probabilities could be obtained from small scale psychological experiments. However, for the remaining part of this paper we shall limit ourselves to fully deterministic individual dynamics. Our goal is to show, on the grounds of a simple social network that even in the deterministic case, the microscopic, agent-to-agent interactions lead to complex social configurations.

In this paper we shall also consider two modes of communication between agents. In the first mode, only one message is sent from the sender to the recipient (‘single message’ mode). This is the situation described in the previous paragraphs and Table 4. After reacting to a message, the recipient agent turns its attention to other messages, authored by different agents. Such situation would be typical for e-mails or the Internet discussion fora involving many users.

In the second mode, both agents exchange the messages, switching the roles of sender/receiver, disregarding all other messages, until a stable configuration of the states of this pair emerges. To show that such stability is achieved, we present, in Table 5, the evolution of states in such exchanges, with the final, stable state shown in boldface. This mode would be denoted as ‘full conversation’ one, because the communication unit is now a conversation between the two users. In the full conversation mode, the two agents who ‘talk’ among themselves turn their attention to other messages only after the reach the final stable state. This mode may correspond to person-to-person meetings in real life or to dedicated Internet chats. Similarly to the case of the single message exchange, the full conversation mode may be described by a transition table containing the final states for the pair of agents, depending on the initial states of the agent starting the conversation and its partner (Table 6).

**Table 5:** Results of subsequent exchanges of messages in a conversation between the ‘Sender’ and the ‘Recipient’.

(CMM,CMM) 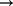 **(CMM,CMM)**
(CMM,C00) 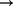 (C00,CMM) 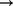 **(CMM,CMM)**
(CMM,CPP) 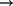 (CPP,C00) 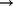 (C00,CPP) 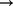 **(CPP,CPP)**
(CMM,AMM) 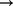 (AMM,CMM) 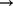 **(CMM,CMM)**
(CMM,A0M) 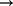 (A0M,CMM) 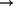 (CMM,C00) 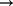 **(CMM,CMM)**
(CMM,A0P) 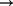 (A0P,AMM) 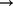 **(AMM,A0P)**
(CMM,APP) 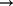 (APP,A0M) 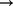 **(A0M,APP)**
(C00,CMM) 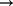 **(CMM,CMM)**
(C00,C00) 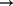 **(C00,C00)**
(C00,CPP) 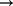 **(CPP,CPP)**
(C00,AMM) 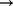 (AMM,C00) 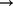 **(C00,AMM)**
(C00,A0M) 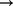 (A0M,C00) 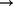 **(C00,A0M)**
(C00,A0P) 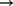 (A0P,C00) 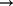 **(C00,A0P)**
(C00,APP) 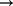 (APP,C00) 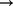 **(C00,APP)**
(CPP,CMM) 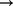 (CMM,C00) 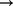 (C00,CMM) 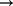 **(CMM,CMM)**
(CPP,C00) 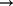 (C00,CPP) 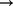 **(CPP,CPP)**
(CPP,CPP) 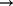 **(CPP,CPP)**
(CPP,AMM) 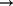 (AMM,A0P) 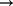 **(A0P,AMM)**
(CPP,A0M) 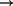 (A0M,APP) 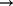 **(APP,A0M)**
(CPP,A0P) 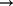 (A0P,CPP) 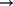 (CPP,C00) 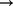 (C00,CPP) 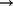 **(CPP,CPP)**
(CPP,APP) 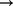 (APP,CPP) 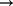 **(CPP,CPP)**
(AMM,CMM) 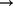 **(CMM,CMM)**
(AMM,C00) 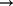 (C00,AMM) 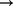 **(AMM,C00)**
(AMM,CPP) 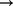 (CPP,A0M) 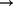 (A0M,APP) 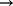 (APP,AOM) 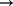 **(A0M,APP)**
(AMM,AMM) 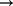 **(AMM,AMM)**
(AMM,A0M) 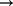 (A0M,AMM) 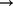 **(AMM,A0M)**
(AMM,A0P) 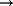 (A0P,AMM) 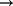 **(AMM,A0P)**
(AMM,APP) 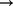 (APP,A0M) 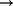 **(A0M,APP)**
(A0M,CMM) 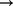 (CMM,C00) 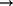 **(CMM,CMM)**
(A0M,C00) 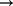 (C00,A0M) 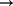 **(A0M,C00)**
(A0M,CPP) 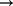 (CPP,A0M) 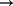 (A0M,APP) 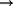 **(APP,A0M)**
(A0M,AMM) 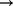 (AMM,A0M) 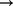 **(A0M,AMM)**
(A0M,A0M) 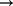 **(A0M,A0M)**
(A0M,A0P) 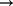 (A0P,A0M) 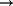 **(A0M,A0P)**
(A0M,APP) 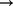 (APP,A0M) 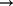 **(A0M,APP)**
(A0P,CMM) 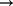 (CMM,A0P) 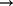 (A0P,AMM) 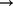 **(AMM,A0P)**
(A0P,C00) 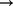 (C00,A0P) 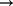 **(A0P,C00)**
(A0P,CPP) 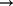 (CPP,C00) 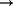 (C00,CPP) 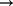 **(CPP,CPP)**
(A0P,AMM) 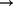 (AMM,A0P) 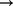 **(A0P,AMM)**
(A0P,A0M) 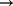 (A0M,A0P) 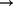 **(A0P,A0M)**
(A0P,A0P) 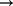 **(A0P,A0P)**
(A0P,APP) 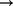 (APP,A0P) 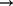 **(A0P,APP)**
(APP,CMM) 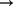 (CMM,A0P) 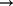 (A0P,AMM) 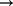 (AMM,A0P) 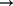 **(A0P,AMM)**
(APP,C00) 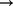 (C00,APP) 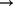 **(APP,C00)**
(APP,CPP) 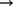 **(CPP,CPP)**
(APP,AMM) 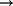 (AMM,A0P) 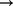 **(A0P,AMM)**
(APP,A0M) 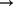 (A0M,APP) 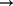 **(APP,A0M)**
(APP,A0P) 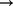 (A0P,APP) 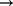 **(APP,A0P)**
(APP,APP) 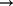 **(APP,APP)**

In each subsequent pair we have reversed the order, to reflect the changes of roles. Stable configurations are in boldface.

**Table 6:** Matrix of final states of two agents resulting from a full conversation between the agent starting the conversation (sender of the first message) and the second agent.

Second	Starting agent						
Agent	CMM	C00	CPP	AMM	A0M	A0P	APP
	CMM	**CMM**	**CPP**	**CMM**	**CMM**	**A0P**	**APP**
CMM	CMM	CMM	**CPP**	**CMM**	**CMM**	**AMM**	**A0M**
	CMM	C00	CPP	AMM	A0M	A0P	APP
C00	**CMM**	**C00**	**CPP**	**C00**	**C00**	**C00**	**C00**
	CMM	**CPP**	**CPP**	**AMM**	**A0M**	**CPP**	**CPP**
CPP	**CMM**	**CPP**	**CPP**	**A0P**	**APP**	**CPP**	**CPP**
	CMM	C00	**APP**	**AMM**	**A0M**	**A0P**	**APP**
AMM	**CMM**	**AMM**	**A0M**	**AMM**	**AMM**	**AMM**	**A0M**
	CMM	C00	**APP**	**AMM**	**A0M**	**A0P**	**APP**
A0M	**CMM**	**A0M**	**A0M**	**A0M**	**A0M**	**A0M**	**A0M**
	**AMM**	**C00**	**CPP**	**AMM**	**A0M**	**A0P**	**APP**
A0P	A0P	A0P	**CPP**	**A0P**	**A0P**	**A0P**	**A0P**
	**AMM**	**C00**	**CPP**	**AMM**	**A0M**	**A0P**	**APP**
APP	**A0P**	**APP**	**CPP**	**A0P**	**APP**	**APP**	**APP**

Any of the two may change its state during the dialogue. Boldface indicates agent states changed due to the encounter.

Tables 4 and 6 are subtly different. In both, most of the states (shown in normal typeface) remain unchanged: neither a single message nor a full conversation would change the state of any agent in the pair. Where changes occur, they may affect the information and/or the emotional state. On the level of the interaction between the two agents, the model is fully deterministic, but allows more complex scenarios than models using only relationships between opinions of the two interacting agents.

## Results

### Deterministic population dynamics

Before we analyze the full model including the information/emotion control variables, we shall consider the cumulative effects of multiple encounters on a specific agent. In this case we are interested in answering a question whether encountering multiple messages dominated by one opinion (e.g. 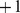) would lead the agent in question to adapt this opinion? The answer depends on the emotional state of the agent and the messages. Calm agents would be convinced by calm messages (either immediately or via intermediate C00 state). But encountering agitated messages promoting opposite opinion turns the agent into the agitated state, in which the responsiveness is smaller and the agent remains, obstinately, with its initial opinion, no matter how many contrary messages it receives. There are social situations where such obstinacy is present, but there are also ones where the perception of majority opinion actually leads people to align their views. An extension of the proposed model allowing the use in such environments shall be discussed in the concluding remarks.

We turn now to discuss global dynamics of a “society” of agents using the proposed individual interactions. As already noted, the proposed model allows very flexible structure of social connections over which the agents interact. Using the message/conversation between pairs of agents picture does not require a specific choice of the social network. Various topologies found in real world systems, such as fixed hierarchies, scale-free or small world can be used as the communication pathways. In all the simulations in this paper we shall limit ourselves to the simplest social network topology, in which all agents are connected to each other. This is obviously too simplistic with respect to most real situations, but our motivation is to look for the effects of the individual dynamics, maximally simplifying the underlying social structure. As such, the results presented below should be treated as indication of possible phenomena allowed by the information/emotion interplay rather than simulations of specific social systems.

In the beginning, we shortly review the behavior of the ‘trivial’ model where only non-emotional states are possible. Depending on the communication mode (single message or full conversation) such system shows very simple behavior. If we consider a ‘society’ comprising of 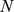 agents, characterized by the initial ratios of agents in specific states 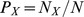, where 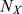 is the initial number of agents in state X (X = CMM, C0M,…) then we may look for stable configurations as the agents interact among themselves, with the evolution given by difference equations. In this paper we assume that in this system, all the agents communicate, and that we are looking at a continuous limit in which each agent reads and reacts to many messages.

If we assume that initial composition of the society (given by 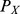) is known we can deterministically calculate the evolution of these values. Even before any calculations are performed, we notice that some starting conditions lead to closed, unchanging situations. For example, if we limit our analysis to purely calm society (the case of 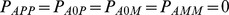), it is possible determine the difference equations containing only calm states for both communication modes, single message and full conversation. In the full conversation case, the stable solution requires 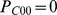 and any combination of 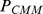 and 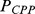 fulfilling the normalization condition 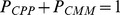 is allowed. In fact the sum 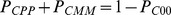 evolves in time as 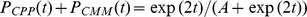, where 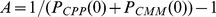. The difference between 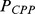 and 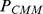 evolves following the same functional form 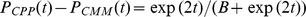, where 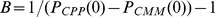.

For the single message case without agitated states, there are only three fixed solutions for 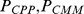 and 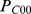. Two of them form asymmetric attractors, with whole society committed to one of the opinions: 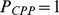, 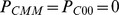 or 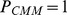, 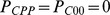. The third fixed point is the symmetric one 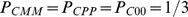, but it is a saddle point and as such not stable under small perturbations.

Summarizing the case of model without emotion: the full conversation mode leads to disappearance of the uninformed/unopinionated agents and a simple form of final total opinions depending on starting conditions. For the single message mode the final distribution is even simpler: full consensus is achieved, in which the population supports either one or the second opinion.

Returning to the model including both calm and agitated agents, the single message mode results in the set of difference equations 1a–1g:
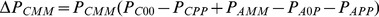
(1a)

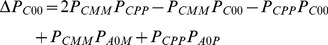
(1b)

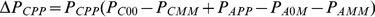
(1c)

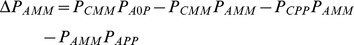
(1d)

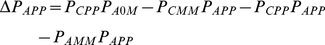
(1e)

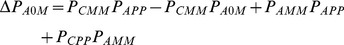
(1f)

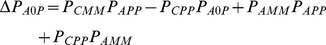
(1g)


The corresponding set of difference equations for the full conversation mode is 2a–2g:
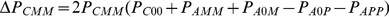
(2a)

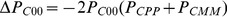
(2b)

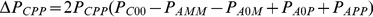
(2c)

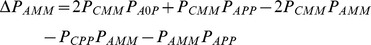
(2d)

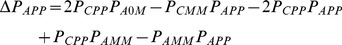
(2e)

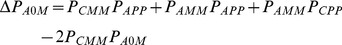
(2f)

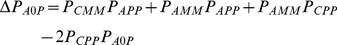
(2g)


All the values of 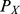 must be between 0 and 1. These equations are complemented by the normalization condition.
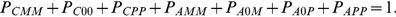
(3)


Because there are now 6 independent variables, description of the fixed points and stable solutions is quite complex. Simple examination of the tables 4 or 6 indicates that certain combinations of 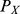 values would remain unchanged trivially, i.e. when the contacts between the agents lead to no change in their status. This happens for example in a population comprising uniformly of agents of a single state, but also in more complex situations such as any combination of 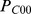, 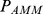, 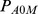 and 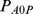 provided that 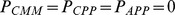 (and symmetrically, when 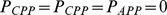). While these distributions remain unchanged under contacts between the users, some of them are not stable in the sense of reaction to small admixtures of other agent states. For the **single message communication mode** the stable configurations are characterized by the following relationships between the 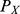 values:
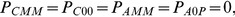
(4a)

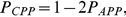
(4b)

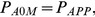
(4c)

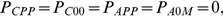
(5a)

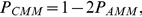
(5b)

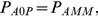
(5c)where 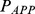 or 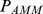 play a role of free variables, limited by conditions 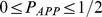 or 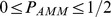. Two additional stable combinations are rather general



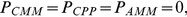
(6a)

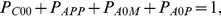
(6b)

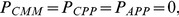
(7a)

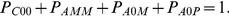
(7b)


When the starting distribution of the different agent states is uniformly distributed over the simplex 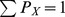, the four stable solutions (4–7) are found with relative frequencies of 47%, 47%, 3% and 3%. The configurations (4) and (5) are interesting in sociological terms. They describe the society where there are no agents without a decided opinion, and where the majority is split into a calm part and an agitated part, the latter exactly balanced by the same number of the minority agents. The minority group is wholly in the agitated state, without enough information to support their opinions, yet ‘obstinately’ holding their opinions. Within the constraints given by these two stable configurations, all possible values of average opinion and emotion are possible.

In the **full conversation mode**, the stable solutions (4) and (5) remain valid, and the solutions (6) and (7) are replaced by a new one. This final configuration has most of the 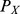 values (except 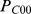) different from zero. A simple algebraic form is obtained choosing 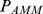 and 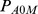 as the independent variables:
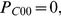
(8a)

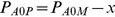
(8b)

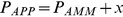
(8c)

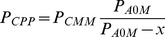
(8d)

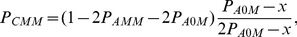
(8e)where 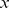 is obtained from a solution of quadratic equation 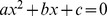, in which 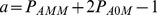, 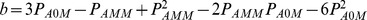 and 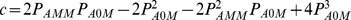.

In a special case, when 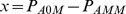 this reduces to:
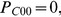
(9a)

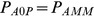
(9b)

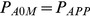
(9c)

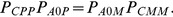
(9d)


### Finite size effects

**Figure 2:**
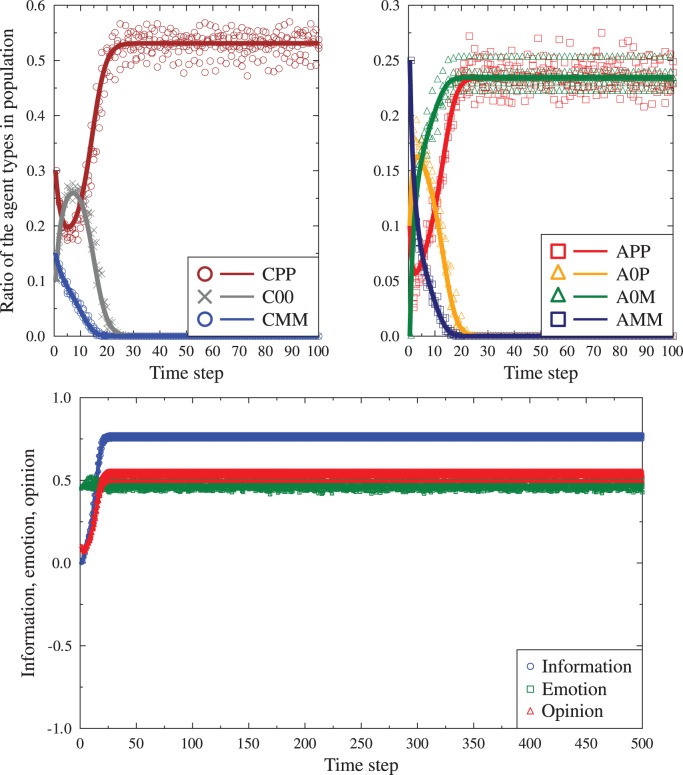
Time evolution of the ratios of agents 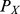 and the average emotion, information and opinion for the single message mode. The starting conditions are: 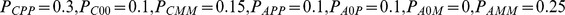. Continuous lines are solutions to the set of difference equations (1), points are examples of finite size (2000 agents) simulations. Top panels: 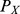 values (the evolution for calm and agitated agents has been separated for visibility). Bottom panes: the associated evolution of global average emotion, information and opinion values.

**Figure 3:**
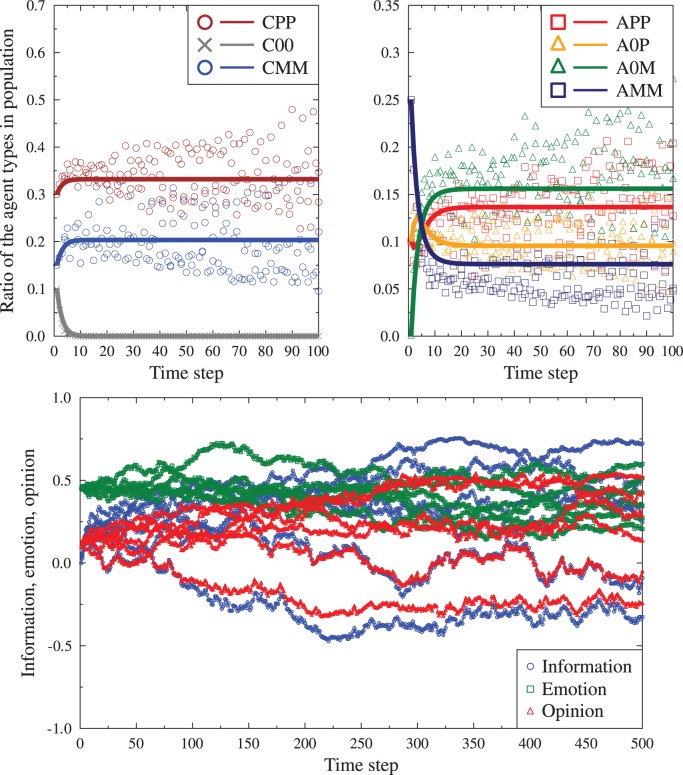
The same data as in **Figure 2**, for the same starting conditions, but for the full conversation mode. Not only are the final values different but also the level of noise given by the finite size simulations is much greater.

The difference equation sets (1) and (2) can be solved numerically for any starting conditions. We should bear in mind that for the purposes of using the information/emotion seven state model in agent based simulations, the number of messages received by each agent should be finite (and realistic in comparison to real life situations). Thus the distribution of the states of the messages received by a particular agent might deviate from the global population distributions. The resulting changes of the agent states would be dictated by local conditions individual history and lead to evolution differing from the solutions of the equations sets (1) and (2). Such deviations are, in our opinion, not a deficiency of the model, but rather a feature allowing to mimic non-deterministic properties of human societies, where individual encounters may influence group behavior on large scales. To check the importance of such finite scale effects we have performed typical agent based simulations, using finite group sizes (between 1000 and 3000 agents) and the same any-to-any communication possibility. In other words, the social network was fully connected. At a single ‘time tick’, each agent read a single message (taken from another, randomly chosen agent). After the reading phase the agents adjusted globally their states to new ones, driven by either the ‘single message’ or the ‘full conversation’ mode. This process was repeated for up to 500 ‘time ticks’. The resulting evolution of the averages of the information, emotion and opinions were recorded, together with the ratios of agents in each state 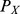. Figures 2 and 3 compare examples of such evolution with the corresponding deterministic evolution following the equations from the previous section. The first observation is that the finite size simulations result in more noisy evolution, but generally follow the form of the deterministic equations. We note that in some cases the evolution of individual 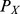 is nonmonotonic, with significant flows of agents between the different states, especially in response to the first 2–20 read messages. After this period the deterministic solutions typically stabilize, while the simulations show smaller or greater amount of noise due to individual history of the system.

**Figure 4:**
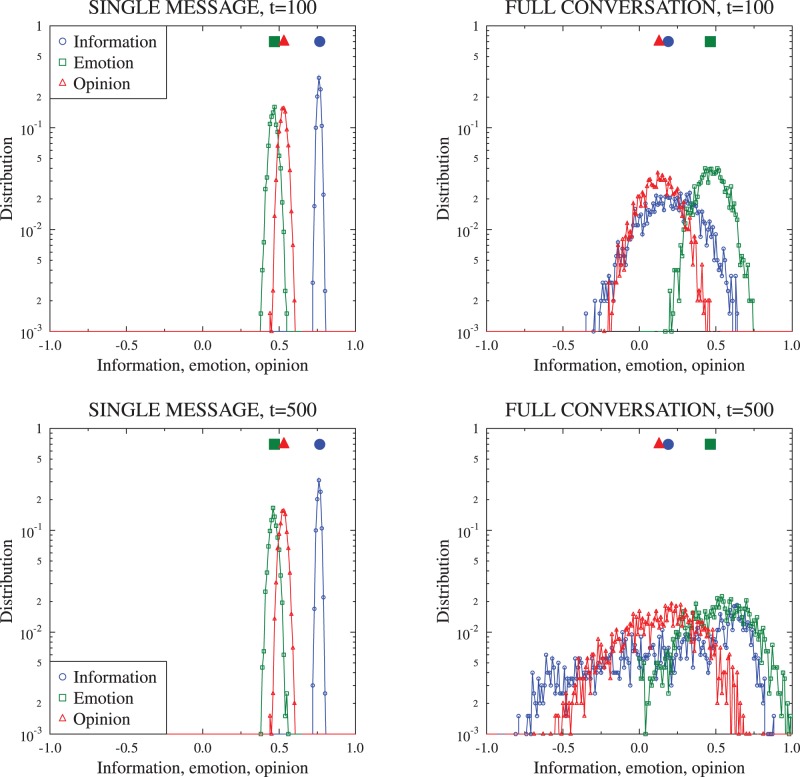
Comparison of distributions of average emotion, information and opinion values obtained during finite size simulations. The same set of initial conditions as for the Figure 2 has been used; statistics are gathered after 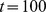 and 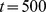 time steps (100/500 messages read by each agent) from 2000 simulation runs. Large scale symbols near the top indicate values from deterministic calculations (equation sets (1) and (2)).

**Figure 5:**
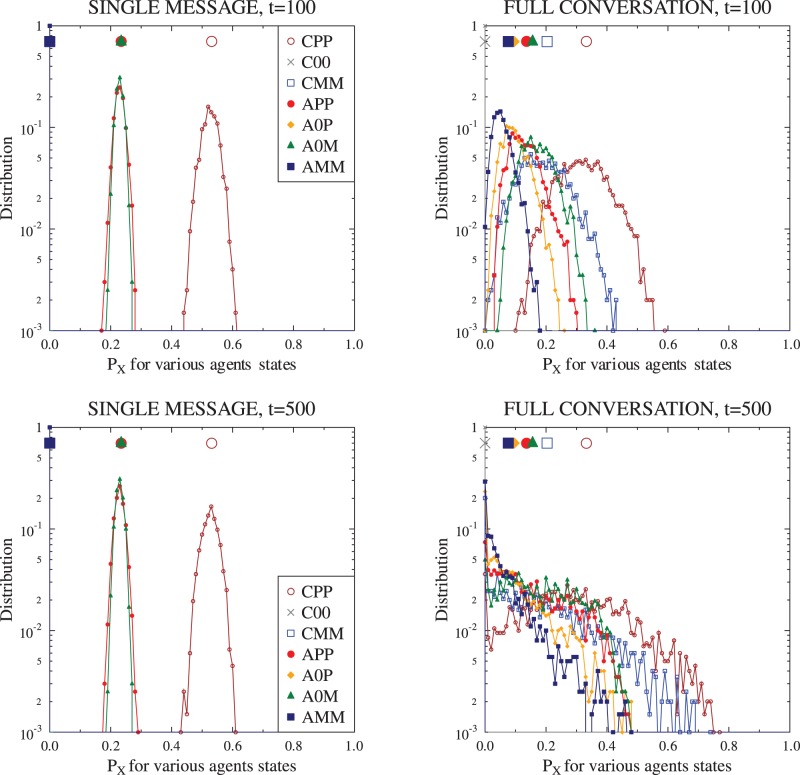
Comparison of distributions of relative occupation ratios 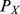 for each agent state obtained during finite size simulations. The same set of initial conditions as for the Figure 2 is used; statistics are gathered after 100 and 500 time steps. Large scale symbols near the top indicate values from deterministic calculations (equation sets (1) and (2)).

Depending on the initial 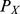 values, the ‘noise’ is smaller or greater. Figures 4 and 5 present comparison of the ‘final’ values from deterministic model with distributions of information, emotion and opinion averages as well as the 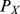 values after 100 and 500 ‘time ticks’. We note that for many values of the starting conditions, the distributions of the final values of 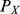 are well described by the Gaussian function, but there are exceptions, where the final distributions are markedly asymmetric.

For some initial values of the agent states distributions, the two communication modes lead to similar behavior, but for others the resulting states are vastly different, as shown in Figures 3–5. The existence of stable configurations mixing most of the agent states (with the exception of the calm, uninformed and unopinionated C00 state) indicates that the model is rich enough to describe the complex social situations.

We note that in addition to the finite size effects one should also consider the finite time effects. The number of messages sent out or received by a person may be, depending on the studied environments, quite small. Thus the effects of the messages on the population evolution may differ from the stable ones discussed above. For example, a typical e-mail environment involves usually less than a few tens of e-mails devoted to a single topic per person, sometimes only a few. As a result, the resulting opinion distribution may be given by a transient rather than the stable value.

### External influences

An interesting property of the proposed model is the natural way in which external influences may be treated. Thanks to the message-based communication, the presence of media or marketing/propaganda efforts may be treated as additional messages in the pool ‘read’ by the agents. These messages may differ among themselves in emotional and informational content allowing to reproduce diversity of media found in real social systems. Such flexibility is not possible in the traditional, spin-based models, where the most common way of introducing external influences is via ‘magnetic field’ analogy.

To illustrate this capability we have chosen a scenario in which a propaganda campaign favoring one point of view is used in a society that initially leans towards the opposite view. Such situation offers not only theoretical but also practical interest. The simulations are started with the following values of the agent states distribution: 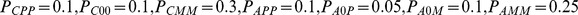. The average opinion of this starting configuration is thus 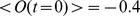. The media messages are assumed to represent either the CPP or the APP combination of emotion/information/opinion. Their frequencies, relative to the number of agents (and therefore to the number of agent generated messages) are 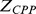 and 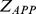. We note that the effect of the media messages is always treated in the ‘single message’ mode, as there is no possibility of ‘talking back’.

**Figure 6:**
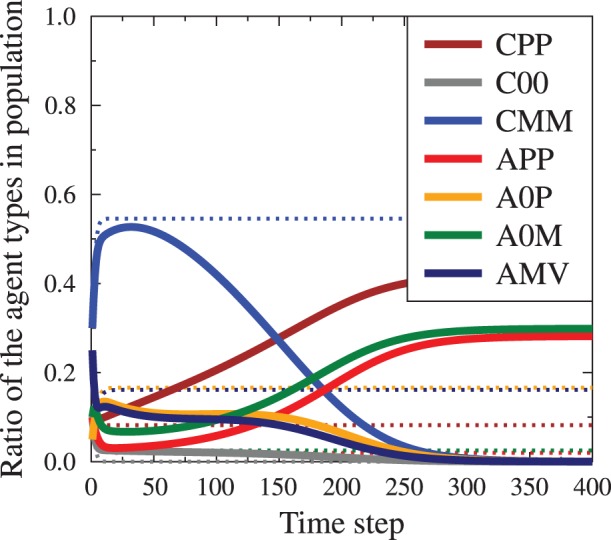
Time evolution of society under external pressure, for the full conversation mode. Dashed lines indicate the evolution of the system without the media messages, the solid lines with the presence, given by 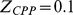, 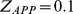. The starting average opinion is 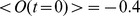. The final, stable average opinion without the media influence is 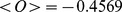, while in the presence of the media it changes to 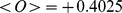, signifying major opinion change in the population.

In the full conversation mode the evolution of the society is relatively straightforward. As shown in Fig. 6 the presence of the propaganda at first slows down the trend of initial CMM majority growth and eventually reverses it, with the final dominance of CPP agents. The timescale of this transition depends on the 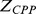 and 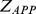 values.

**Figure 7:**
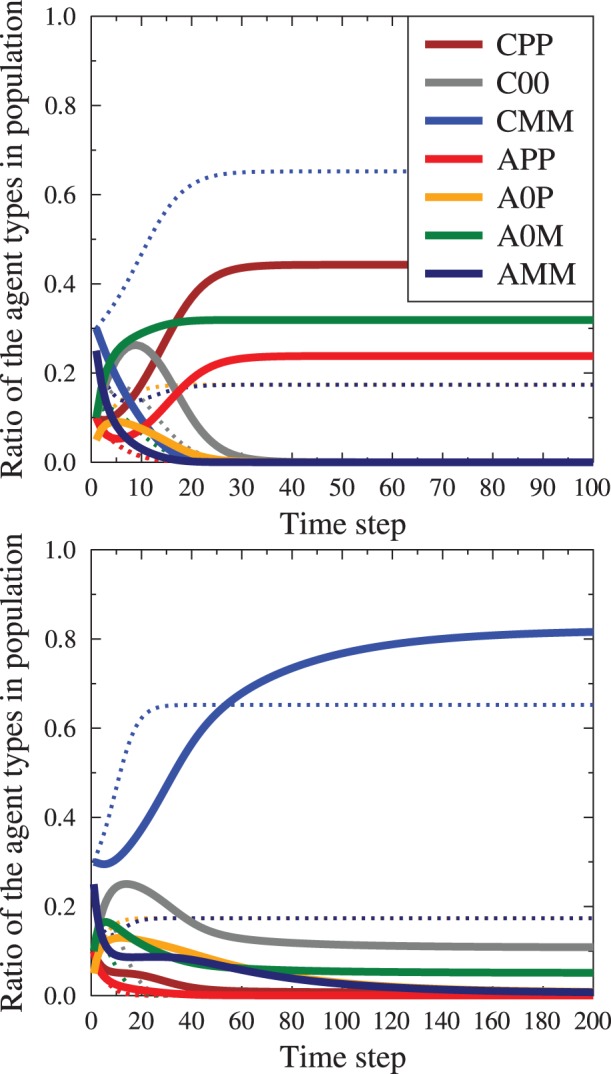
Time evolution of a society under external pressure, for the single message mode. Top panel: situation with strong enough external influence (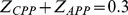), convincing the agents to form a majority in CPP state. Bottom panel: ‘paradoxical’ state (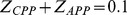), where the presence of the propaganda actually strengthens the starting majority which opposes the propaganda. Note the difference in time scales. Dashed lines show the evolution with the same starting composition but no external messages. The starting average opinion is 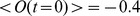. The final, stable average opinion without the media influence is 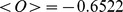. For the strong external influence of 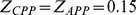 it changes to 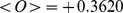, signifying major opinion change in the population. For weak external influence 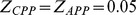, a paradoxical state with the average opinion 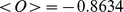 results.

In the ‘single message’ mode the evolution is more complex (Fig. 7). If the ratio of the calm external messages (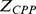) is large enough the CPP agents rather quickly dominate the society. But for low 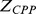 values a paradoxical effect is observed: the stable configuration which evolves has a majority of the CMM agents! In fact, if only calm media messages are present, the final configuration has only calm agents and the relative values of final 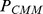, 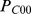 and 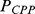 are given by solutions of the equations 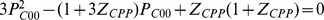, 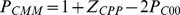, 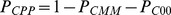. Microscopically the mechanism is a rather complex interplay of transfers of agents to a calm state by media messages that agree with their position, and once calmed, the agents are capable of accepting the viewpoint of the CMM majority (if it is large enough compared to 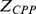).

**Figure 8:**
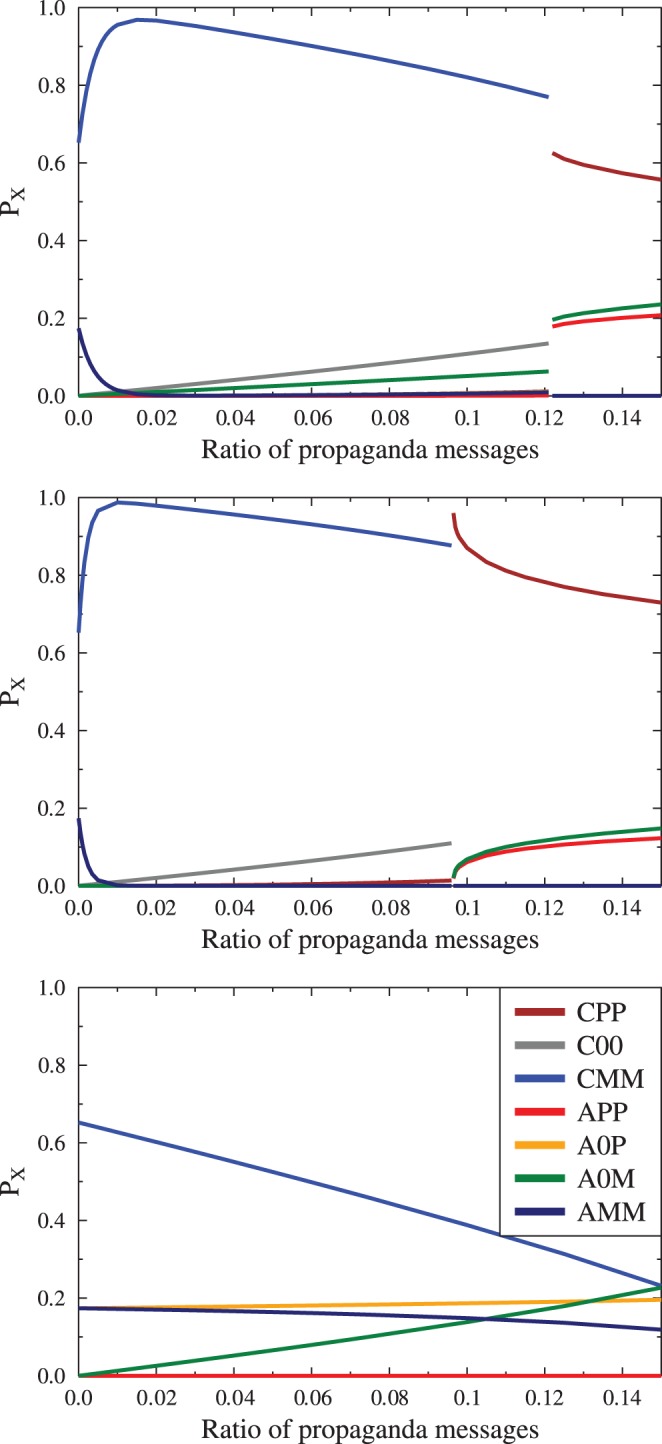
Examples of dependence of the distribution of final composition of society (agent states 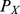) on the ratio of propaganda messages to the total number of agents. Bottom panel: pure 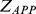, which shows a gradual increase of the number of agitated agents without significant change of opinion. The mixed case (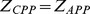, top panel) and the pure 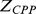 case (middle panel) show a sudden transition between the ‘paradoxical’ state (in which the propaganda presence strengthens the opposite view and the case where propaganda actually succeeds in convincing a majority of the agents). This transition occurs for certain value of the 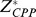, which depends on the initial composition of 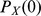. The transition is related to the existence of a long lived metastable state (see Fig. 9), which suggests a phase transition due to symmetry breaking by the external influence of propaganda messages.

**Figure 9:**
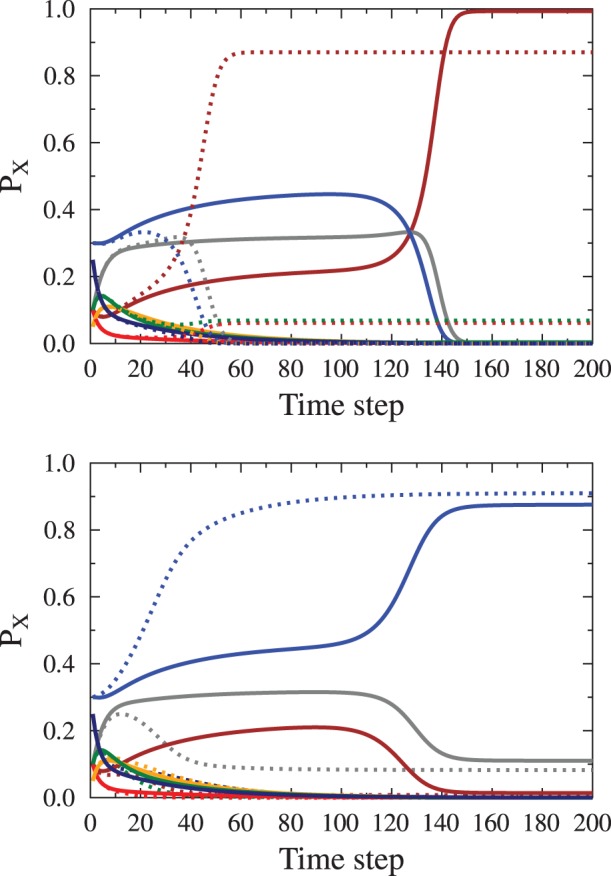
Examples of time evolution of agent states occupation ratios 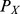 as function of the ratio of calm propaganda messages to the total number of agents 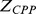. Bottom panel: evolution slightly below the transition point 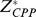, top panel – slightly above 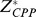. Solid lines show evolution for values very close to the transition point; dashed lines, for comparison, show the evolution far from the critical value. Color codes as in the previous figures. The presence of a metastable state (seen between time step 20 and 120) is an indicator of symmetry breaking by the single sided external propaganda. with the same starting conditions 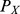, characterized by the average opinion 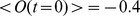 the final oiutcomes are drastically different. Below 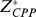, the average opinion is 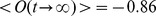 (paradoxical state, where positive propagande leads to an increased negative opinions), above the threshold it is 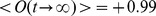 (the propaganda takes full effect, leading to almost full consensus). The average opinion during the metastable state varies a little but it is close to 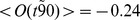, so that there is some effect of the positive propaganda. By getting closer to the 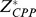 value the lifetime of the metastable state may be arbitrarily extended.

As the two types of the final state are very much different, we have investigated the behavior of the system when 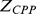 and 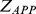 values are changed. Figure 8 compares the final values of 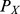 as function of the total 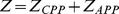 in three cases: pure 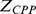, mixed case (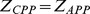) and pure 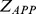. In the last case we observe gradual change of distributions, with increasing presence of the agitated agents. In the cases where calm external arguments we observe a sudden transition between the two states, the paradoxical one and the situation where propaganda convinces large part of the society. The transition point is characterized by very long existence of a metastable state as shown in Fig 9. This metastable state is characterized by the persistence of a significant number of calm, unopinionated agents (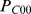), whose numbers fall dramatically when the metastable state changes into the final configuration. The presence of such metastable states is an indicator that there is a symmetry breaking in the system, introduced by the presence of single sided propaganda messages. The critical value 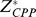 (see figures 8,9) depends on the initial distribution of agent's information and emotions 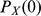. We note that the presence of symmetry breaking is very interesting, but remains to be confirmed if it would still be present in situations when the simple wholly connected social topology is replaced by more realistic ones, such as scale free or hierarchical network.

## Discussion

The model presented in this work is aimed at providing a simple microscopic (agent-to-agent) dynamics of influences, extending beyond models depending on opinion only. Additionally, we have focused on message based communication model. There are several reasons for proposing such a combination.

First, the introduction of the two emotion levels allows to describe the situation where two distinct opinions are possible for the same set of the control variables. Such decoupling of opinion from the control role allows, on an individual agent level, significant resistance to the social pressures (coming from single agents, groups or external influences) and provides the ground for hysteresis phenomena, in similarity to continuous cusp catastrophe models. The advances in applications of catastrophe theory (together with attempts to derive the parameters of the cusp surface), such as the already mentioned works [41], [48], [50]–[53], indicate that this may be an important direction. The discrete approach we propose has fewer degrees of freedom but still preserves the key features of the continuous model. This simplifies the simulations and allows a more straightforward mapping of the agent based and real worlds. Using the discretized values, the distribution of opinions and emotions is much easier to determine. For example one could make practical use of the growing number of datamining algorithms aimed at automated analysis of records of Internet based communications. Another way of checking the validity of assumptions and improving them, already mentioned, would be via small scale laboratory experiments.

The second intention was to base the interaction between the agents on more realistic, ‘social’ process than the one used in the majority of the sociophysical approaches, which owe their origins to spin-spin interaction descriptions. The use of messages (which reflect the current state of their author) corresponds directly to many social situations: e-mail networks, Internet discussions, modern Internet based social networking environments. In many of such environments it would be possible to base the computer simulations on the actual properties of the studied environment (social network topology and dynamics, frequency of the messages, effects of moderation on their emotional content etc.). This would allow a direct solution of the problem of matching the simulation parameters and the social environment. Moreover, the advances in datamining techniques, allowing analysis of the content of the messages (emotional and informational) could allow testing of the basic assumptions of the proposed microscopic dynamics. An additional benefit of the message based approach is that it allows to estimate the realistic measures for the ‘simulation time flow’. This is typically measured and reported in Monte Carlo steps, which are very difficult to map into the time flow in real life. Here such comparison is natural and given by comparisons of the rates of message creation/reception.

Even in the traditional environments, where contacts between people are not based on distinct, separate messages, the proposed approach may have some value. The ‘full conversation’ mode was introduced with this goal in mind, where face-to-face encounters between the agents may lead to simultaneous, reciprocal adjustments of their states.

The third characteristic of the model is the broad spectrum of results, from the microscopic level of contacts between single agents to the macroscopic evolution of the composition of the simulated social group. Our original motivation was to describe the behavior of users of an Internet discussion forum, within a single discussion thread, to model the changes of emotions and opinions. But the model can also be applied to more complex environments. As we demonstrated, even in the case of simple, fully connected social network there are many possible transient and final states. The possibility that a single agent would retain its opinion despite the interactions with other agents, even if they would form a perceived majority, naturally leads to the possibility of the existence of minority groups. These groups might be very stable without the need to introduce mechanisms such as cutting off the social links to people of different opinion [62] (without excluding such mechanisms, which may or may not be present in a concrete social situation). We note here that the microscopic mechanism does not depend on any assumptions regarding the social network and may be studied in short range, scale free or random networks; both static and dynamically changing.

The last feature of the proposed model is the ease of introducing external pressures, such as marketing campaigns, propaganda, or mass media. Within the traditional, ‘spin-based’ opinion models, the external influence was usually modeled as equivalent to a magnetic field. This resulted in a problem of properly scaling the ‘strength’ of the external influence, in comparison with the agent-agent interactions. Moreover, such approach did not allow to treat effectively situations such as the existence of two media sources, one trusted and one distrusted by the different groups of agents, phenomenon widely recognized in social studies. In our approach marketing activities, propaganda efforts and media are treated as additional sources of messages received by the agents and treated accordingly to their emotion/information states.

The specific applications of the proposed approach could include: analyses of dialogues between pairs of discussion fora users, with occasional influence of other users. Most likely a probabilistic interpretation of the reaction should be used in such a case. The goals of such simulations would be to recreate the emotion evolution, clusters of calm/agitated states and correlations of emotions/opinions. Another example of application is a model of a closed society reaction to news. The social network should be taken from observations (e.g. gathered from datamining a discussion forum or from advanced techniques such as the scalable real-time data gathering via RFID, resolving face-to-face social interactions, as described by Cattuto et al. and Stehle et al. [63], [64]) and then fed with a new and possibly controversial information. The stirring information might or might not be repeated. A specially interesting case would be the study of reactions to competing media messages. The goal would be a parallel model of social and media polarization.

The model presented in this work, with its deterministic interactions between identical agents is, of course, too simple to describe real human motivations, decision processes and interactions. Still, our goal was to show, that inclusion of a nonlinear information/emotion interplay can lead to nontrivial social situations.

One way of improving the model, without changing the basic conceptual framework, would be to replace the assumption of identical agents interacting deterministically with a model in which each agent would be described by a set of parameters that would determine probabilities of its actions and reactions. Such a set could include:

- set of probabilities of changing the agent's information state, {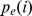}, replacing the deterministic transitions of information state (given by Tables 1 and 2) by adjustable dynamics, depending on agent and message emotional state. Changing these parameters would allow to flexibly model the resistance to influences of the other agents or to propaganda. Such flexibility would allow to give greater weight to information accumulated by an individual in the past (e.g. from previously read messages). Moreover even a small probability of change could lead to accepting the opposite information is the agent is exposed to sufficiently large number of messages supporting such view, this would answer the problems mentioned in the opening paragraphs of the section describing the deterministic dynamics;
- probability of sending a message, 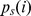, in a given timeframe, allowing to distinguish differences in activity of the agents;
- probability of reading/receiving a message (which might come from another agent or from the propaganda pool), 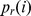;
- probability of the arousal of emotions in a calm agent due to encounter with calm but contrary opinion, 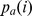. The arousal probability enters into the system dynamics when a calm message is received by a calm agent with opposing view. The agent may then, instead of accepting the information and turning into a calm, information balanced and un-opinionated state, keep its information and opinion, but turn into the corresponding agitated state, for example from CMM to AMM when encountering an CPP message. In other words, the probability 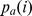 measures the agent irritability or irrationality. Its inclusion allows creation of the emotionally aroused agents from initially calm society, a feature which is absent in the deterministic model described in detail in this work.
- probability of calming down 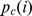, when an agitated agent, who does not participate in any discussion in a given time step, i.e. does not send out nor read any message changes into non-agitated state.
- Generally, the probability of calming down (decreasing emotion from 1 to 0 for the agent 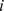), within a single timeframe could lead to the following situations:
- with probability 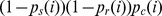 - the agent is inactive and calms down. Specifically, the transitions would be: AMM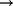 CMM, APP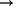 CPP, A0M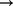 C00, A0P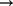 C00.
- with probability 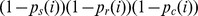 - agent is inactive and remains in the unchanged state;
- with probability 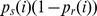 - the agent sends a message but does not receive/read anything, therefore remains in the unchanged state;
- with probability 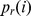 - the agent reads a message and, depending on the relationship between the message information/emotion and its own state may change its state to a new value.

Calming down allows for emotion relaxation in societies where communication is relatively infrequent. Coupled with the fact that calm agents are easier to convince, this model extension allows a more realistic treatment of real social situations.

The respective distributions of these probabilities that might be used to model a social system should be derived for the specific situation from social and psychological research; for example the activity distributions might be obtained from network analysis while the arousal/calming probabilities could be obtained from psychological profiling.

Other possible expansions of the model, going beyond the scope of the current report, might include: memory for specific agent-agent relationship; allowing some differentiation of credibility and trustworthiness of some sources of messages, for example leadership status for agents or preferred status of certain media messages and avoidance of others (selective attention [65], [66]) and different lifetimes and impacts of individual messages. The inclusion of such memory effects (e.g [32]) and variable impact (e.g. [67]) is possible within the model when the agents and messages are “individualized” instead of being given the same properties. Still, as our goal was to provide a flexible framework and point out its potential value for social studies, we decided to keep the initial model as simple as possible, sacrificing psychological accuracy as these enhancements complicate the model, they should be introduced cautiously, when the goal is to describe specific social situations where such distinctions are observed and judged important.
